# Circulating tumor cells in metastatic breast cancer patients treated with immune checkpoint inhibitors – a biomarker analysis of the ALICE and ICON trials

**DOI:** 10.1002/1878-0261.13675

**Published:** 2024-07-08

**Authors:** Nikolai Kragøe Andresen, Andreas Hagen Røssevold, Elin Borgen, Cecilie Bendigtsen Schirmer, Bjørnar Gilje, Øystein Garred, Jon Lømo, Marius Stensland, Oddmund Nordgård, Ragnhild Sørum Falk, Randi R. Mathiesen, Hege G. Russnes, Jon Amund Kyte, Bjørn Naume

**Affiliations:** ^1^ Department of Clinical Cancer Research Oslo University Hospital Norway; ^2^ Department of Cancer Immunology, Institute for Cancer Research Oslo University Hospital Norway; ^3^ Institute of Clinical Medicine University of Oslo Norway; ^4^ Department of Pathology Oslo University Hospital Norway; ^5^ Department of Hematology and Oncology Stavanger University Hospital Norway; ^6^ Department of Chemistry, Bioscience and Environmental Technology University of Stavanger Norway; ^7^ Oslo Centre for Biostatistics and Epidemiology Oslo University Hospital Norway; ^8^ Department of Oncology Oslo University Hospital Norway; ^9^ Department of Cancer Genetics, Institute for Cancer Research Oslo University Hospital Norway; ^10^ Faculty of Health Sciences Oslo Metropolitan University Norway

**Keywords:** breast cancer, circulating tumor cells, immune checkpoint inhibitors, liquid biopsy

## Abstract

Immune checkpoint inhibitors (ICIs) have been introduced in breast cancer (BC) treatment and better biomarkers are needed to predict benefit. Circulating tumor cells (CTCs) are prognostic in BC, but knowledge is limited on CTCs in the context of ICI therapy. In this study, serial sampling of CTCs (CellSearch system) was evaluated in 82 patients with metastatic BC enrolled in two randomized trials investigating ICI plus chemotherapy. Programmed death‐ligand 1 (PD‐L1) expression on CTCs was also measured. Patients with ≥ 2 CTCs per 7.5 mL at baseline had gene expression profiles in tumor suggestive of increased T‐cell activity, including increased tumor inflammation signature (TIS) in both triple‐negative (*P* = 0.010) and hormone receptor‐positive (*P* = 0.024) disease. Patients with luminal A BC had higher CTC levels. The association between CTC status and outcome was most apparent 4 weeks into therapy. PD‐L1 expression in CTCs was observed in 6/17 CTC‐positive patients and was associated with inferior survival. In conclusion, our study indicates that CTC numbers may inform on tumor immune composition, as well as prognosis. These findings suggest a potential of using CTCs as an accessible biomarker source in BC patients treated with immunotherapy.

## Graphical abstract

In this explorative biomarker analysis, we assessed serial sampling of circulating tumor cells (CTCs) with CellSearch in two randomized trials testing immune checkpoint inhibitors (ICIs) in metastatic breast cancer. Our data demonstrate a prognostic potential of CTCs, most apparent 4 weeks into ICI therapy. Furthermore, higher CTC counts were associated with increased markers of tumor immune activity.

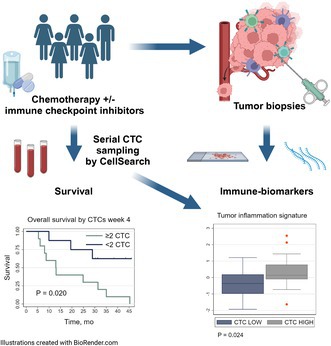

**Abbreviations**
BC: breast cancer
CTCs: circulating tumor cells
ECOG: Eastern Cooperative Oncology Group
EOT: end of treatment
EPCAM: epithelial cell adhesion molecule
FFPE: formalin‐fixed, paraffin‐embedded
HER2^−^
: human epidermal growth factor receptor 2‐negative
HR^+^
: hormone receptor‐positive
ICIs: immune checkpoint inhibitors
IHC: immunohistochemistry
IQR: interquartile range
OS: overall survival
PD‐L1: programmed death‐ligand 1
PFS: progression‐free survival
TILs: tumor‐infiltrating lymphocytes
TIS: tumor inflammation signature
TME: tumor microenvironment
TNBC: triple‐negative breast cancer

## 1 Introduction

The evolving new therapies in breast cancer have improved the outcomes for patients with both early and advanced breast cancer (BC). However, the treatments are only effective for some of the patients, at the cost of increased complexity and toxicity for all. There is a need for biomarkers, beyond classical breast cancer subtyping, to identify patients likely to benefit. Immunotherapy is a treatment option with such unresolved biomarker needs. Antibodies blocking negative regulators of T‐cell activation (immune checkpoint inhibitors; ICIs) have demonstrated benefit in combination with chemotherapy for patients with metastatic triple‐negative breast cancer (mTNBC) expressing programmed death‐ligand 1 (PD‐L1) [1, 2]. Today, antibodies blocking the PD1/PD‐L1 axis combined with chemotherapy are standard of care for both localized high‐risk TNBC and PD‐L1‐positive mTNBC [1, 2, 3]. For the non‐TNBC population, the role of immunotherapy is still under consideration with a number of ongoing clinical trials [4, 5].

A large proportion of patients do not benefit from the addition of immunotherapy to chemotherapy. Furthermore, immune‐related adverse events caused by off‐target immune activation are common [1, 2], some of these life‐threatening [6]. In the need for tools to guide the clinician's treatment decisions, primary and metastatic tissues have been used to search for biomarkers, so far with none established beyond PD‐L1 expression. An alternative option to tumor tissue analysis is a ‘liquid biopsy’ approach, including circulating tumor DNA and circulating tumor cell (CTC) analyses [7, 8].

The CellSearch system isolates CTCs from peripheral blood by immuno‐magnetic enrichment of epithelial cell adhesion molecule expressing (EpCAM^+^) cells [9, 10]. The assay has been validated in multiple settings and found to provide highly reproducible results across laboratories [11, 12]. The prognostic value of CTC enumeration by CellSearch in mBC was first demonstrated by Cristofanilli and colleagues two decades ago [13]. The presence of ≥ 5 CTCs per 7.5 mL blood before initiation of new treatment indicated both inferior progression‐free and overall survival (PFS, OS). Lower cutoffs for CTC positivity have also been shown to give prognostic information. The prognostic value of CTCs has later been confirmed in large pooled analyses of both mBC [14] and early‐stage breast cancer [15]. In addition to pre‐treatment assessments, early CTC dynamics in response to therapy is predictive of outcome with both chemo‐ and endocrine therapy [14, 16, 17, 18], while highly prognostic, randomized trials evaluating treatment decisions based on CTCs have failed to show a survival benefit for the overall study population [18, 19]. However, in the randomized STIC CTC trial CTC‐driven use of chemotherapy improved both PFS and OS among hormone receptor‐positive (HR^+^)/human epidermal growth factor receptor 2‐negative (HER2^−^) mBC patients considered ‘clinical low risk’ and otherwise recommended endocrine therapy [20, 21].

Tumor PD‐L1 expression measured by immunohistochemistry (IHC), either assessed on tumor cells, infiltrating immune cells or a combination is the main biomarker for selecting patients to PD1/PD‐L1 inhibition in mTNBC [1, 2]. Importantly, samples are often archival and heterogeneity in PD‐L1 status between both assays and metastatic sites are high [22, 23]. The CellSearch system enables CTCs to be analyzed for expression of PD‐L1 as a liquid biopsy, and this may counter tumor heterogeneity and also provide tumor sampling without the need of a new tissue biopsy. Data from published studies suggest frequent PD‐L1 expression on CTCs across different metastatic tumor types [24, 25, 26, 27]. It is not known how CTC profiles relate to the immune composition of the tumor microenvironment (TME) or whether PD‐L1 expression on CTCs has any predictive potential for the effect of immunotherapy.

Here we report data on serial CTC sampling in mBC patients randomized to chemotherapy or chemotherapy plus ICI in the two phase II trials ALICE (NCT03164993) [28] and ICON (NCT03409198) [29]. We aimed to explore the prognostic value of CTCs in patients receiving ICI therapy compared to conventional therapy, including its potential in early response assessment. We also explored associations between CTC profiles and immune biomarkers from matched tumor samples including PD‐L1 expression, tumor‐infiltrating lymphocytes (TILs), and immune gene expression signatures.

## 2 Materials and methods

### 2.1 Patients and study therapy

Samples were collected from subjects enrolled in the two randomized phase II trials ALICE (TNBC) [30] and ICON (hormone receptor‐positive; HR^+^) [31] evaluating anthracycline‐based chemotherapy alone or in combination with ICI in mBC. The studies were approved by the Regional Committee for Medical Research Ethics south‐east Norway (ID: #14195 & #2017/1283) and the Norwegian Medicines Agency. Written informed consent was obtained from all patients, and the trials were conducted according to the Declaration of Helsinki and guidelines of Good Clinical Practice.

All patients had confirmed HER2^−^ mBC with either triple‐negative (ALICE) or HR^+^ (ICON) histology. Other key eligibility criteria included Eastern Cooperative Oncology Group (ECOG) performance status of 0 or 1, a disease‐free interval of minimum 12 months from prior anthracyclines or cyclophosphamide in the (neo‐) adjuvant setting to recurrence and no more than one previous line of chemotherapy in the metastatic setting. HR^+^ patients had no limitations on previous endocrine or targeted therapy.

The chemotherapy regimen was identical in both trials. Patients were randomized in a 2 : 3 ratio to either pegylated liposomal doxorubicin 20 mg·m^−2^ i.v. every 2nd week and cyclophosphamide 50 mg po daily (every 2nd 2‐week cycle) alone (chemo‐only), or in combination with ICI. TNBC patients received the chemotherapy in combination with atezolizumab 840 mg i.v. every 2nd week (atezo‐chemo). HR^+^ patients received the chemotherapy combined with ipilimumab 1 mg·kg^−1^ i.v. every 6th week and nivolumab 240 mg i.v. every 2nd week (ipi/nivo‐chemo). HR^+^ patients stopping chemo‐only were offered ipilimumab and nivolumab without chemotherapy in a separate cross‐over cohort (ipi/nivo‐only) [31].

Radiological assessments were performed every 8th week from randomization for the first 12 months and every 12th week thereafter. Efficacy data were recorded with RECIST 1.1 [32] as the primary method and iRECIST [33] as the secondary method.

### 2.2 Circulating tumor cell sampling and enumeration

The subset of patients enrolled at the study sites Oslo University Hospital and Stavanger University Hospital were analyzed for CTCs. Samples were collected from March 2019 to February 2023. If possible, sampling in each cohort was performed at up to 4 time points: baseline, 4 weeks, 6 months, and/or at the end of treatment (EOT) visit. Whole blood was collected into 10 mL CellSave tubes (Menarini Silicon Biosystems, Bologna, Italy) and analyzed within 96 h at the Oslo University Hospital research lab. CTC enumeration was performed using the CellSearch system (Menarini Silicon Biosystems). The CellSearch system is a semi‐automatic system that captures EpCAM expressing cells with immunomagnetic selection in samples of 7.5 mL whole blood [10]. For the standard CTC analysis, the isolated EpCAM‐positive (EPCAM^+^) cells are then labeled by immunofluorescent staining for cytokeratins (CK8, CK18, and CK19) with the phycoerythrin fluorochrome (PE), nuclear staining by DAPI and the leukocyte antigen CD45 with the allophycocyanin fluorochrome (APC) [9]. Isolated EpCAM^+^ elements negative for CD45 with CK expression and nuclear staining are manually confirmed as CTCs by a set of strict morphological criteria. Starting January 2020, CTC enumeration was done with the CXC kit (Menarini Silicon Biosystems; see below).

### 2.3 Circulating tumor cell PD‐L1 assessment

From the first analysis in March 2019 until January 2020, samples were processed with the standard CTC kit. Thereafter, samples were analyzed with the CXC kit, a research only kit containing a cytokeratin‐fluorescein (FLU) reagent instead of the standard cytokeratin‐PE reagent enabling both CTC enumeration and staining with another PE‐conjugated antibody [9]. For assessment of PD‐L1 expression, the PE‐conjugated extracellular domain‐specific rabbit IgG anti‐PD‐L1 antibody D8T4X (Catalog #71319, Cell Signaling Technology, Danvers, MA, USA) was used in a 1 : 30 dilution with an integration time of 0.2 s. The CXC/D8T4X assay was first tested on whole blood from four healthy donors (HD) confirming a variable PD‐L1 signal in some white blood cells. Then, samples from HD were spiked with the PD‐L1‐positive breast cancer cell line MDA‐MB‐231 (ATCC®HTB‐26, RRID:CVCL_0062) and the PD‐L1‐negative cell line BT‐474 (ATCC®HTB‐20; RRID:CVCL_0179). The PD‐L1 status of the MDA‐MB‐231 and BT‐474 cell lines was first confirmed by flow cytometry (Fig. S1A,B). CTC PD‐L1 staining intensity was scored on a 0–2 scale, categorized as: no signal in the PE‐channel (0), dim signal but not stronger than the brightest signal also observed in the PD‐L1‐negative BT‐474 cell line (+1), brighter signal than any CTC in the negative cell line (+2; Fig. S1C). Of hundred CTCs assessed from HD samples spiked with the PD‐L1‐positive MDA‐MB‐231 cell line, 98 CTCs had a +2 PD‐L1 staining intensity, while 2 CTCs were negative (0). Out of 59 CTCs isolated from samples spiked with the BT‐474 cell line, a dim signal (+1) in the PE‐channel was observed in 33 of 59 CTCs. For data analysis, CTC PD‐L1 status is grouped as positive (+2) vs. negative (< 2). A threshold of PD‐L1 expression in ≥ 10% of the CTCs was set to reduce the risk of false‐positive samples in patients with high CTC counts. Fourteen patients had two CellSave tubes collected at the same time point to compare enumeration variability with the CTC and CXC kit (Fig. S2).

### 2.4 Assessment of tissue PD‐L1, tumor‐infiltrating lymphocytes, and immune gene expression

Formalin‐fixed, paraffin‐embedded (FFPE) tumor samples from primary or metastatic lesions were obtained for comparative analyses between CTC profile at baseline and tumor PD‐L1 expression by the SP142 assay, tumor‐infiltrating lymphocytes (TILs), and immune gene expression signatures. In patients with more than one pre‐treatment sample available for analysis, the most recent tumor sample was used.

PD‐L1 expression by IHC on archival tumor samples was available from all patients with baseline CTC samples (*n* = 55). The VENTANA PD‐L1 (SP142) assay (Roche Diagnostics, Rotkreuz, Switzerland) was used for PD‐L1 expression analysis. Samples were scored by two senior breast cancer pathologists according to the Interpretation Guide for Triple‐Negative Breast Carcinoma [34] assessing PD‐L1 expression on tumor‐infiltrating immune cells, with infiltration of ≥ 1% of the tumor area as a cutoff for positivity.

The presence of TILs was assessed in hematoxylin‐ and eosin‐stained slides from all patients with a baseline CTC sample (*n* = 55). TILs were assessed in a study screening biopsy if sufficient material, otherwise in the most recent archival biopsy available. The abundance of infiltrating lymphocytes within the tumor borders was scored on a 0–3 scale. For data analysis, the TIL score was grouped as low (score 0/1) or high (score 2/3).

Tumor gene expression was analyzed on bulk RNA isolated from archival FFPE sections with the nCounter® Breast Cancer 360™ (BC360) assay (NanoString Technologies, Seattle, WA, USA). The RNA isolation procedure and initial data analysis has been previously described [30]. The BC360 assay identifies expression of 776 single genes with an output including 42 gene expression signatures and the intrinsic breast cancer subtype. The calculation of gene expression signatures and the molecular subtype was performed by NanoString Technologies. Eighteen of the signatures are related to immune function, including the tumor inflammation signature [35]. In patients with a baseline CTC profile, gene expression data were available from 53 of 55 patients with a combination of metastatic samples (*n* = 31) and primary lesions (*n* = 22).

### 2.5 Study objectives and statistical considerations

CTC samples were collected in both clinical trials as a part of the protocol‐defined exploratory objective of identifying novel biomarkers for clinical response to ICI combinations. Sampling was not sufficiently funded from study initiation. Thus, not all patients and time points were sampled. In addition to enumeration data, we aimed to evaluate the prognostic value of PD‐L1 expression on CTCs and compare CTC profile with immune‐related biomarkers from the clinical trials. In patients with a missing week 4 or EOT sample, with disease progression or censoring directly (< 1 month) following the 4 week time point, the week 4 result was used as EOT (5 patients) and EOT result as week 4 (2 patients). For descriptive statistics, continuous data are presented as median with interquartile range (IQR) and categorical data as frequencies with percentages. The Wilcoxon rank‐sum test was used for group comparison of CTC enumeration data, the *t*‐test for gene expression data, and Fisher's exact test for categorical data. Survival analyses are based on the Kaplan–Meier method with comparison between groups presented as hazard ratios (HR) with 95% confidence intervals and the log‐rank test. Follow‐up time has been calculated by the reverse Kaplan–Meier method. All *P* values are two‐sided with *P* values < 0.05 considered statistically significant. Adjustment for multiple testing was not performed. stata se v.18.0 (StataCorp LLC, College Station, TX, USA) and r v.4.1.2 were used for statistical analyses.

## 3 Results

**Fig. 1:**
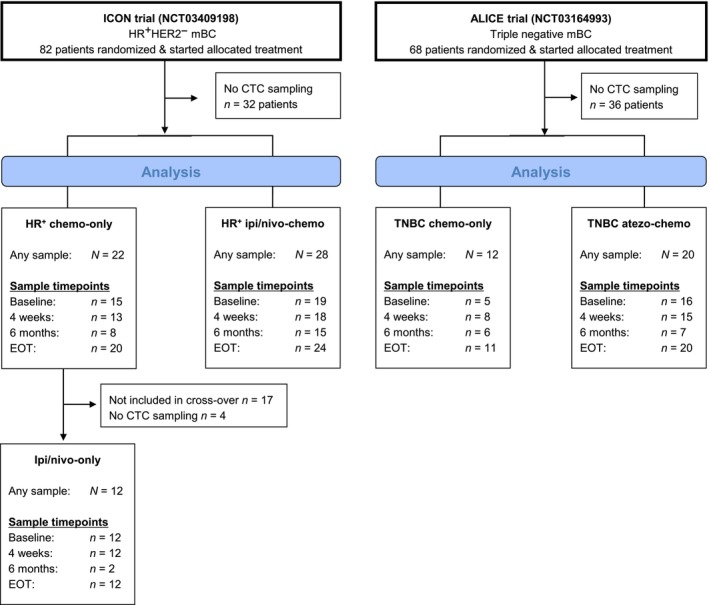
Overview of treatment cohorts and CTC sampling. For the purpose of the current study, CTC data from the 6 months of sampling in the main treatment cohorts have not been included in this analysis. atezo, atezolizumab; CTCs, circulating tumor cells; EOT, end of treatment; HER2^−^, human epidermal growth factor receptor 2‐negative; HR^+^, hormone receptor‐positive; ipi, ipilimumab; mBC, metastatic breast cancer; nivo, nivolumab; TNBC, triple‐negative breast cancer.

CTC samples were collected from 82 patients (*n* = 32 TNBC, *n* = 50 HR^+^) between March 2019 and February 2023. A flow diagram of the treatment cohorts and CTC sampling is presented in Fig. 1. Median follow‐up time for patients with baseline samples was 39.9 months (IQR 31.2–43.4). Baseline demographics and disease characteristics are shown in Table S1. Time from diagnosis of stage IV disease was different in the TNBC and HR^+^ populations reflecting both biology and previous lines of endocrine treatment. Lung metastases were more common in the TNBC population, while disease dissemination to liver and bone was more frequent in the HR^+^ patients.

**Fig. 2:**
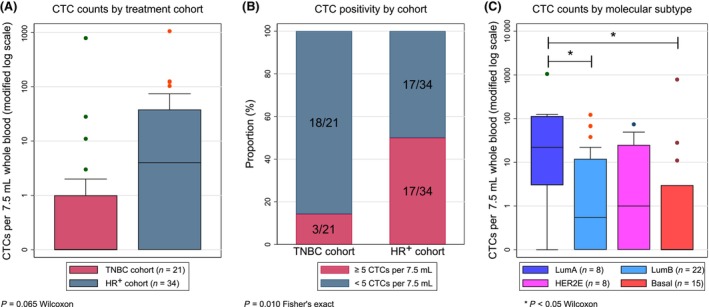
Baseline CTC distribution by histological and molecular subtype. Panel (A) presents CTC counts at baseline in the TNBC (*n* = 21; left) and HR^+^ (*n* = 34; right) population. Panel (B) presents the distribution of patients in each cohort relative to the ≥5 CTCs per 7.5 mL cutoff at baseline. The distribution of CTC counts by the molecular subtype is presented in panel (C). Subtype classification was based on gene expression analysis of archival FFPE samples. In patients with more than one sample typed, the most recent sample available was used for classification. The boxes in (A) and (C) represent the middle half of the data between the 25th and 75th percentiles, and the line represents the median. CTCs, circulating tumor cells; HER2E, HER2 enriched; HR^+^, hormone receptor‐positive; TNBC, triple‐negative breast cancer.

Baseline CTC samples were available from 55 of 82 patients (67%) with a median CTC count of 0 CTC per 7.5 mL (range 0–783) in the TNBC cohort vs. 4 CTCs per 7.5 mL (range 0–1050) in the HR^+^ cohort (Wilcoxon *P* = 0.065; Fig. 2A). Table S2 shows patient characteristics according to CTC cutoffs of ≥ 5 and ≥ 2 CTCs per 7.5 mL. ECOG performance status 1, shorter interval from time of metastatic disease to inclusion, and increased baseline level of C‐reactive protein (CRP) were associated with CTC positivity in HR^+^ patients for both cutoffs. Only 3 of 21 patients (14%) in the TNBC cohort had baseline CTCs above the ≥ 5 CTCs per 7.5 mL cutoff, compared to 17 of 34 patients (50%) in the HR^+^ cohort (Fisher's exact test *P* = 0.010; Fig. 2B). The baseline CTC distribution varied by molecular subtype (Fig. 2C), with the highest median counts in patients with luminal A subtype (22 CTCs per 7.5 mL, IQR 3–114) and lower counts in both the basal‐like (0 CTC per 7.5 mL, IQR 0–3; Wilcoxon *P* = 0.021) and luminal B populations (0.5 CTCs per 7.5 mL, IQR 0–12; Wilcoxon *P* = 0.047).

### 3.1 Associations between baseline CTC profile and tumor immune biomarkers

Current understanding on how CTCs relate to the immune composition of the TME is limited. Therefore, we analyzed the relationship between the CTC status at baseline and PD‐L1 expression by IHC in archival biopsies (55/55 patients), TILs in screening biopsies (55/55 patients), and the 18 gene expression signatures related to immune function from the NanoString BC360 assay in archival samples (53/55 patients). To circumvent the small number of TNBC patients with ≥ 5 CTCs per 7.5 mL, the ≥ 2 CTCs per 7.5 mL cutoff was chosen for these analyses.

**Fig. 3:**
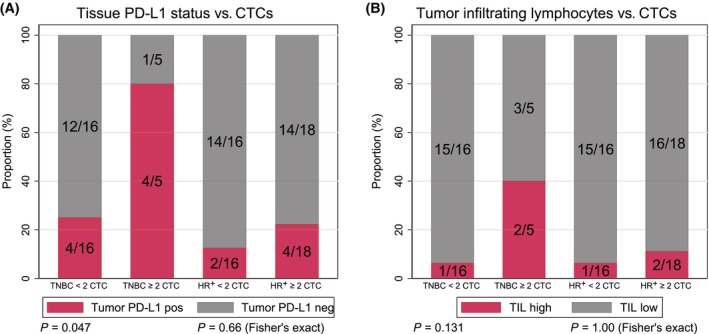
Baseline CTC counts vs. tumor tissue PD‐L1 status and TIL score. Associations between the baseline CTC count (≥ 2 CTCs per 7.5 mL) and tissue PD‐L1 status by IHC and TIL score were analyzed. The bar chart in panel (A) shows the distribution of PD‐L1 status by the SP142 assay in the CTC high and low group for the TNBC (left) and HR^+^ (right) population. The bar chart in panel (B) presents the distribution of TIL score high and low vs. CTC counts for both breast cancer subtypes. PD‐L1 status was analyzed in the most recent archival sample available. The TIL score was assessed in a study screening biopsy if sufficient material, otherwise in the most recent archival biopsy available. CTCs, circulating tumor cells; IHC, immunohistochemistry; PD‐L1, programmed death‐ligand 1; TILs, tumor‐infiltrating lymphocytes.

In the TNBC population, four of five patients (80%) with ≥ 2 CTCs per 7.5 mL at baseline had PD‐L1‐positive biopsies, vs. four of 16 patients (25%) with < 2 CTCs per 7.5 mL (Fisher's exact test *P* = 0.047; Fig. 3A). TNBC patients with increased CTCs also had a higher proportion of TIL high tumors, 2/5 patients (40%) vs. 1/16 patients (6%; Fisher's exact test *P* = 0.131; Fig. 3B). These patterns were not apparent in HR^+^BC patients (Fig. 3A,B).

**Fig. 4:**
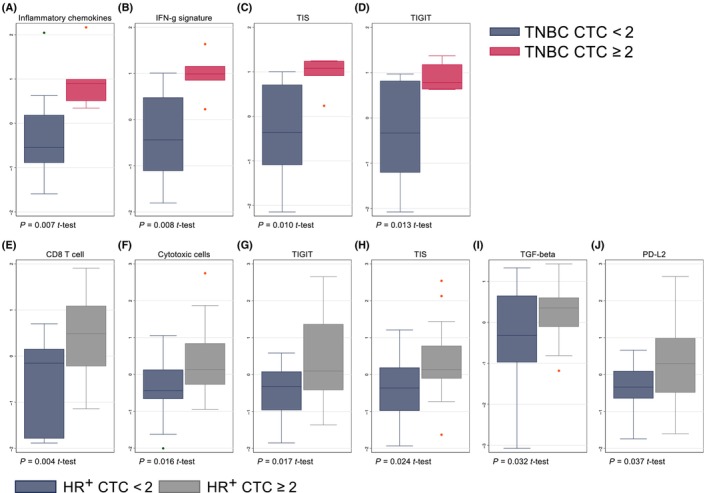
Baseline CTC counts vs. immune‐related gene expression signatures. Panels (A–D) presents the most differentially expressed (*P* < 0.05) immune gene signatures from the NanoString BC360 assay in patients with low CTCs (*n* = 15) vs. high CTCs (*n* = 5) at baseline (≥ 2 CTCs per 7.5 mL cutoff) within the TNBC population. Panels (E–J) presents the most differentially expressed signatures in CTC low (*n* = 15) vs. CTC high (*n* = 18) patients within the HR^+^ population. The boxes represent the middle half of the data between the 25th and 75th percentiles, and the line represents the median. *P* values are calculated with the *t*‐test. CTCs, circulating tumor cells; HR^+^, hormone receptor‐positive; IFN‐g, interferon gamma; PD‐L2, programmed cell death ligand‐2; TGF‐beta, transforming growth factor‐β; TIS, tumor inflammation signature; TNBC, triple‐negative breast cancer.

The BC360 assay includes 42 gene expression signatures. Figure S3A,B shows the differential expression of these signatures in patients with ≥ 2 vs. < 2 CTCs per 7.5 mL. Interestingly, in both the TNBC and HR^+^ cohort, gene signatures related to immune function were among the most differentially expressed. In the TNBC cohort, the mean expression levels of 17/18 measured immune signatures (all but mast cell signature) were numerically higher in patients with ≥ 2 CTCs per 7.5 mL (Fig. S3A). Moreover, in the HR^+^ cohort all 18 immune‐related signatures were numerically higher (Fig. S3B). The data distribution of immune signatures with a statistically significant difference in expression level (*P* < 0.05) is presented by breast cancer subtype in Fig. 4A–J. The most differentially expressed signatures were linked to CD8 T‐cell activity and a T‐helper 1 (Th1) weighted cytokine profile. The tumor inflammation signature (TIS), which is reported to be predictive of responses to PD‐1 blockers in several cancer forms [35], was increased in patients with high CTCs, in both the TNBC and HR^+^ population (Fig. 4C,H).

### 3.2 Survival outcomes by baseline and week 4 CTC counts

CTC levels pre‐treatment and after treatment initiation are prognostic for survival outcomes in mBC patients treated with conventional therapy [13, 14]. However, in the setting of ICI‐based therapy data are limited and the associations presented above highlight potential differences. Thus, we explored the prognostic value of baseline and week 4 CTC status on survival outcomes in all cohorts. PFS and OS were analyzed by both the ≥ 2 CTCs per 7.5 mL cutoff and ≥ 5 CTCs per 7.5 mL cutoff.

**Fig. 5:**
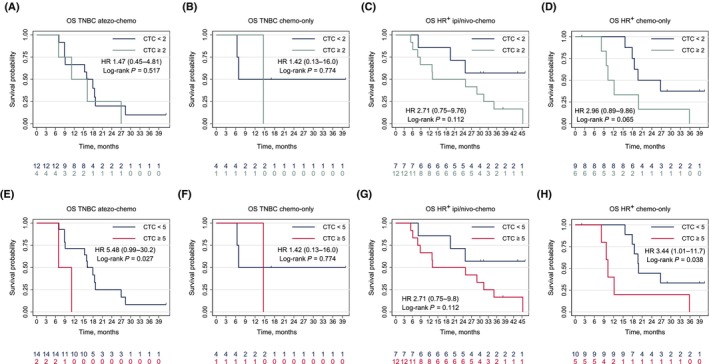
Overall survival by baseline CTC count. The figure presents Kaplan–Meier plots of OS by the ≥ 2 CTCs per 7.5 mL cutoff in each of the four treatment cohorts in (A–D). OS by the ≥ 5 CTCs per 7.5 mL cutoff in each cohort is presented in (E–H). atezo, atezolizumab; CTCs, circulating tumor cells; HR, hazard ratio; HR^+^, hormone receptor‐positive; ipi, ipilimumab; nivo, nivolumab; OS, overall survival; PFS, progression‐free survival; TNBC, triple‐negative breast cancer.

We did not detect PFS differences by baseline CTC counts in any of the treatment cohorts (Fig. S4). In TNBC patients receiving atezo‐chemo, OS did not differ by < 2 vs. ≥ 2 CTCs per 7.5 mL (HR 1.47, 95% CI 0.45–4.81; log‐rank *P* = 0.517; Fig. 5A), while the two patients with ≥ 5 CTCs per 7.5 mL had poor survival (HR 5.48, 95% CI 0.99–30.2; log‐rank *P* = 0.027; Fig. 5E). In both of the two HR^+^ treatment cohorts, patients with increased baseline CTCs had a similar negative OS trend (Fig. 5C,D,G,H).

**Fig. 6:**
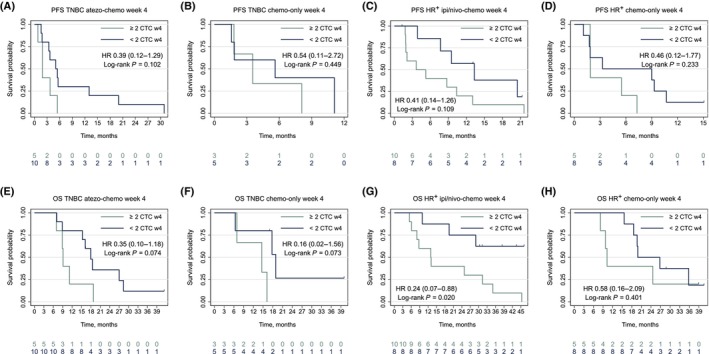
Survival outcomes by week 4 CTC count (≥ 2 CTCs per 7.5 mL). The figure presents Kaplan–Meier plots of survival outcomes by CTCs measured at week 4 by the ≥ 2 CTCs per 7.5 mL cutoff in each of the four treatment cohorts. PFS is presented in panel (A–D), and OS is presented in panel (E–H). atezo, atezolizumab; CTCs, circulating tumor cells; HR, hazard ratio; HR^+^, hormone receptor‐positive; ipi, ipilimumab; nivo, nivolumab; OS, overall survival; PFS, progression‐free survival; TNBC, triple‐negative breast cancer; w4, week 4.

On‐treatment samples were collected at week 4 (median 29 days, IQR 28–30). The proportion with ≥ 2 CTCs per 7.5 mL at 4 weeks was similar in the immune‐chemo and chemo‐only arms both in the TNBC cohort with 33% (5/15) vs. 38% (3/8; Fisher's exact test *P* = 1.00) and in the HR^+^ cohort with 56% (10/18) vs. 38% (5/13) (Fisher's exact test *P* = 0.47). Survival outcomes for all cohorts are presented in Fig. 6. All patients (5/5) with ≥ 2 CTCs per 7.5 mL at week 4 in the TNBC atezo‐chemo cohort had PFS < 6 months and a negative OS trend (HR 0.35, 95% CI 0.10–1.18; log‐rank *P* = 0.074; Fig. 6A,E). In the HR^+^ ipi/nivo‐chemo cohort, < 2 CTCs per 7.5 mL was associated with a numerically better PFS (HR 0.41, 95% CI 0.14–1.26; log‐rank *P* = 0.109; Fig. 6C) and superior OS with 5/8 patients still alive at data cutoff vs. none (0/10) with ≥ 2 CTCs per 7.5 mL (HR 0.24, 95% CI 0.07–0.88; log‐rank *P* = 0.020; Fig. 6G). PFS and OS by the ≥ 5 CTCs per 7.5 mL cutoff are presented in Fig. S5.

As supplementary information, pooled survival analyses for patients in both studies regardless of study therapy are presented in Fig. S6, showing the strongest prognostic survival signal according to CTC status at week 4 (compared to baseline; Fig. S6E–H).

### 3.3 CTC dynamics in response to dual checkpoint inhibition

**Fig. 7:**
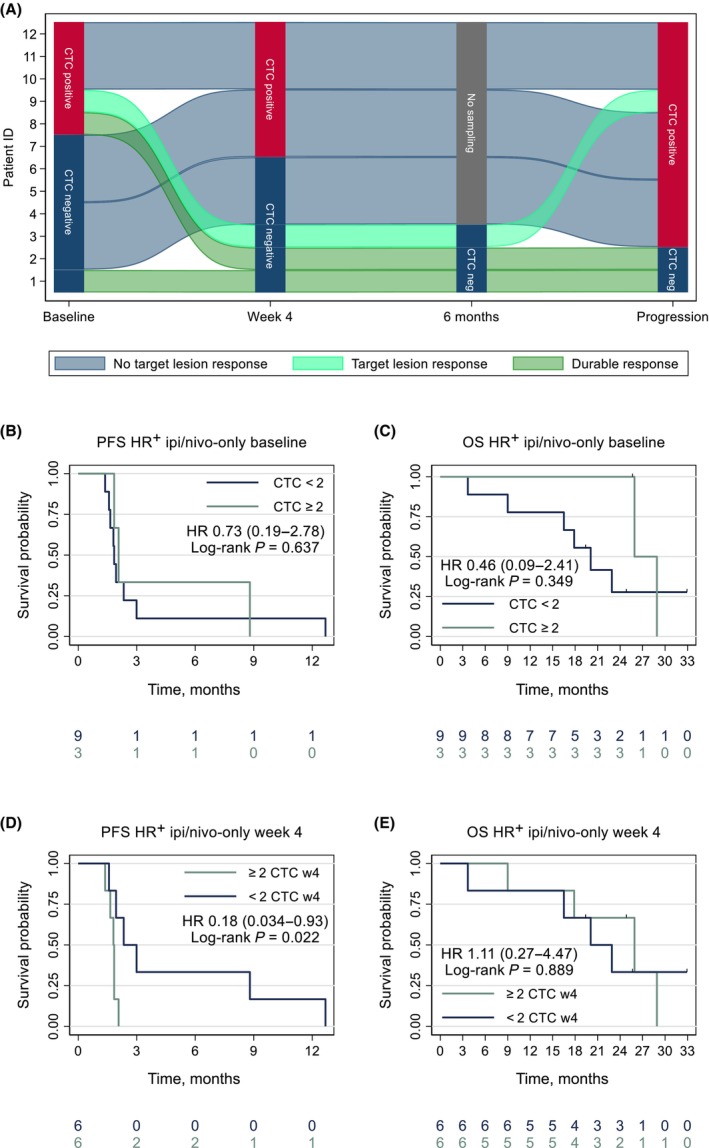
CTC dynamics and survival outcomes in patients receiving ipi/nivo‐only. The diagram in (A) presents the observed CTC dynamics in the HR^+^ ipi/nivo‐only cohort (*n* = 12) with CTC measurements at baseline, week 4, 6 months, and the time of progression. Cutoff for positivity was ≥ 1 CTC. Three patients had measurable target lesion reduction, with two of the three developing durable responses. Panel (B, C) presents Kaplan–Meier plots of PFS and OS in the ipi/nivo‐only cohort by baseline CTCs by ≥ 2 CTCs per 7.5 mL. Panels (D, E) presents Kaplan–Meier plots of PFS and OS by CTC measured 4 weeks into therapy by the ≥ 2 CTCs per 7.5 mL cutoff. CTCs, circulating tumor cells; HR, hazard ratio; HR^+^, hormone receptor‐positive; ipi, ipilimumab; nivo, nivolumab; OS, overall survival; PFS, progression‐free survival; w4, week 4.

The HR^+^ cross‐over cohort treated with ipilimumab and nivolumab after stopping chemo‐only (ipi/nivo‐only; *n* = 12) gives an insight into the pattern of CTC response to immune checkpoint inhibition. A diagram of the observed CTC dynamics under treatment is presented in Fig. 7A. Five out of 12 patients (42%) had detectable CTCs at baseline, while only 3 of 12 patients (25%) had ≥ 2 CTCs per 7.5 mL. Survival was not inferior in the three patients with ≥ 2 CTCs per 7.5 mL at baseline (Fig. 7B,C). Four weeks into therapy, two patients had converted to CTC‐negative status, and both had reduction of target lesions. In total, 3 of 12 patients (25%) had any reduction of target lesions, and 2 of them developed durable responses (> 6 months). PFS and OS by ≥ 2 CTCs per 7.5 mL at week 4 are presented in Fig. 7D,E. Notably, the two responders had no detectable CTCs at both the 6‐month time point and the time of progression (Fig. 7A). Conversely, all (10/10) non‐responders had detectable CTCs at the time of progression (Fisher's exact test *P* = 0.015). Both patients with durable CTC clearance developed new metastatic lesions. The first patient developed bilateral adrenal metastases, which is very uncommon in HR^+^ mBC [36]. The second patient developed multiple brain metastases without any other extracerebral disease activity.

To further explore this pattern of negative samples post‐durable ICI response, survival in HR^+^ patients with disease progression was analyzed by CTC presence at EOT. Two patients in each cohort without progression at EOT were therefore excluded from the analysis. The proportion with negative CTC samples was similar with 23% (5/22) vs. 28% (5/18) in the HR^+^ ipi/nivo‐chemo and HR^+^ chemo‐only cohorts, respectively (Fisher's exact test *P* = 0.73). In the HR^+^ ipi/nivo‐chemo cohort, negative samples were associated with superior OS post‐progression calculated from the EOT time point (HR 8.01, 95% CI 1.04–61.7; log‐rank *P* = 0.018; Fig. S7A). A similar pattern was observed in the ipi/nivo‐only cohort (log‐rank *P* = 0.322), but not among HR^+^ chemo‐only patients (HR 1.27, 95% CI 0.39–4.12; log‐rank *P* = 0.685; Fig. S7B,C). These results were not observed in the TNBC cohorts (data not shown).

### 3.4 PD‐L1 expression on circulating tumor cells

**Fig. 8:**
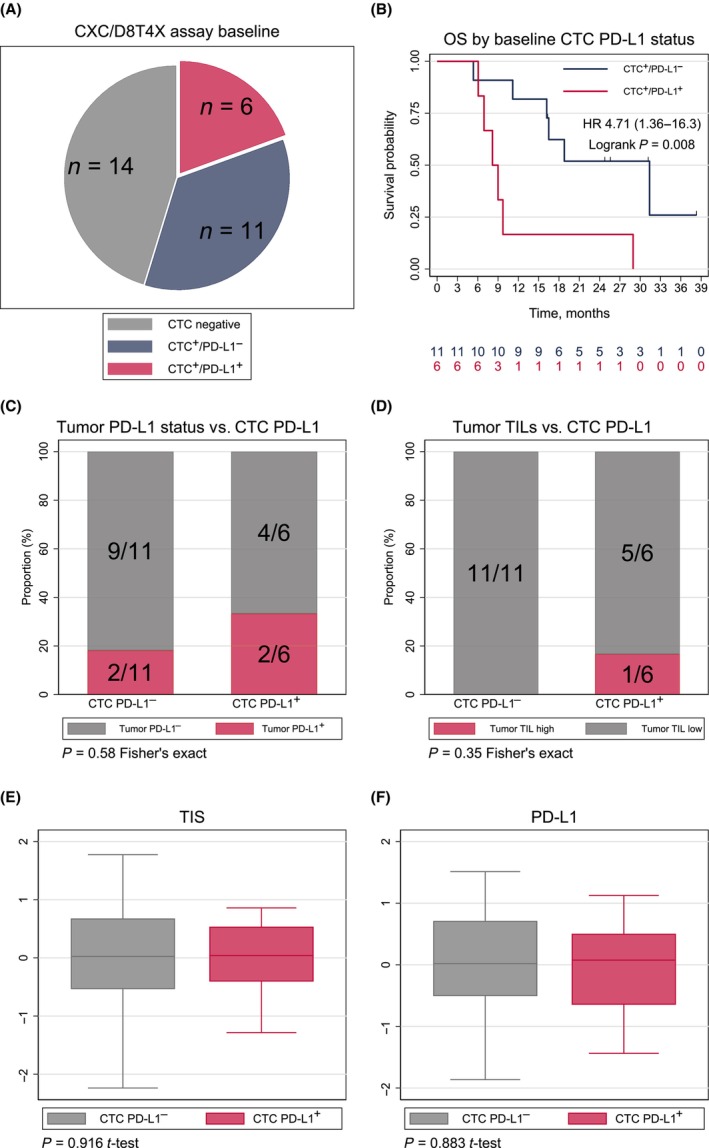
CTC PD‐L1 expression. The diagram in panel (A) presents the 31 patients with baseline samples analyzed with the D8T4X/CXC assay. OS by baseline CTC PD‐L1 status regardless of treatment cohort is presented in (B). The bar chart in panel (C) presents the tumor PD‐L1 status by IHC with the SP142 assay in CTC PD‐L1‐negative (*n* = 11) vs. PD‐L1‐positive (*n* = 6) patients at baseline. Panel (D) presents the proportion with high or low tumor‐infiltrating lymphocytes by CTC PD‐L1 status at baseline. The box plots in (E, F) present the tumor expression levels of TIS and PD‐L1 gene expression signature by CTC PD‐L1 status. The boxes represent the middle half of the data between the 25th and 75th percentiles, and the line represents the median. CTCs, circulating tumor cells; HR, hazard ratio; OS, overall survival; PD‐L1, programmed death‐ligand 1; TILs, tumor‐infiltrating lymphocytes; TIS, tumor inflammation signature.

Thirty‐one patients had baseline samples analyzed with the CXC/D8T4X assay enabling assessment of PD‐L1 expression on CTCs. CTCs were detected in 17 of 31 patients (55%; Fig. 8A). A PD‐L1 signal of any intensity, including the uncertain dim 1+ score also observed in the negative controls, was detected in 14 of 17 patients (82%). With the predefined threshold of a 2+ staining intensity in ≥ 10% of the CTCs, 6 out of 17 patients (35%) were PD‐L1‐positive (PD‐L1^+^; Fig. 8A) with a median count of PD‐L1^+^ CTCs of 1.5 CTCs per 7.5 mL (IQR 1–5). The PD‐L1^+^ proportion was 20% (1/5) in the TNBC and 42% (5/12) in the HR^+^ cohort. PD‐L1^+^ patients had numerically higher total CTC counts with a median of 7.5 CTCs per 7.5 mL (IQR 1–18) vs. 3 CTCs per 7.5 mL (IQR 1–5) in the PD‐L1‐negative CTC patients (Wilcoxon *P* = 0.30).

To assess the biological impact of CTC PD‐L1^+^ status, efficacy outcomes and immune biomarkers were analyzed by CTC PD‐L1 expression. The efficacy analyses were limited by only three patients with PD‐L1^+^ baseline samples receiving ICI. None of the three had an objective response vs. 27% (3/11) in the PD‐L1‐negative CTC population. In a combined analysis of all CTC‐positive patients evaluated for PD‐L1 expression at baseline regardless of study therapy, OS was inferior in the PD‐L1^+^ population (HR 4.71, 95% CI 1.36–16.3; log‐rank *P* = 0.008; Fig. 8B).

How PD‐L1 expression on tumor cells isolated from circulation is related to immune‐related biomarkers from matched tumor samples is still uncertain. To assess this, we compared CTC PD‐L1 status with PD‐L1 expression by the SP142 assay in tumor biopsies, tumor‐infiltrating lymphocytes (TILs), and two NanoString immune gene expression signatures related to T‐cell activity. The PD‐L1‐positive proportion by IHC in tumor tissue was 33% vs. 18% in the CTC PD‐L1‐positive and PD‐L1‐negative populations, respectively (Fisher's exact test *P* = 0.58; Fig. 8C). Only one patient had TIL high status not allowing any informative assessment (Fig. 8D). The tumor inflammation signature and tumor PD‐L1 gene expression had equal levels in the two groups (Fig. 8E,F).

## 4 Discussion

Here we report an exploratory analysis of CTC data from two randomized ICI trials in mBC with extensive biomarker profiles of matched tumor samples. The prognostic value of CTCs in the setting of ICI combinations, including serial sampling of CTCs in a rare cohort of HR^+^ patients treated with ipi/nivo alone, is presented. Our findings support a prognostic potential of CTCs measured 4 weeks into ICI based therapy, similar to what is established for conventional therapy. Beyond this, the small sample size restricts the survival interpretations. Intriguingly, our analyses indicate that CTC profiles are associated with biomarkers of tumor immune activity.

We observe that patients with ≥ 2 CTCs per 7.5 mL at baseline have increased markers of an immune‐activated TME. Importantly, findings are consistent across the two breast cancer subtypes and in both PD‐L1 IHC and gene expression data. This is an exploratory finding that needs to be validated in other cohorts. Currently, there is lack of literature comparing CTC data with the wider tumor immune cell composition. But interestingly, Xue et al. [37] found the presence of CTCs, assessed with a PCR assay, to be associated with an increased number of intratumoral regulatory T cells in mBC. Overall, the TME is known to follow distinct spatial patterns of immune infiltration [38]. Distinct immunophenotypes described as inflamed, excluded, and ignored are also found in TNBC [39]. These spatial phenotypes relate to the underlying mechanisms of tumor immune evasion [38, 40]. Within an inflamed TME, tumor and immune cells have direct cell to cell contact and this phenotype is associated with both PD‐L1 expression and increased expression of gene signatures related to adaptive immunity [39]. Likewise, CTCs in circulation are fully exposed to the immune system. Thus, a hypothesis would be that CTCs arising from a more inflamed TME have developed immune evasive strategies that allow CTCs to escape systemic immunosurveillance in the peripheral circulation.

The TIS gene expression profile was associated with increased CTCs in both TNBC and HR^+^ patients. It contains 18 genes related to the adaptive immune response selected to predict response to PD‐1 inhibition across cancer subtypes [35, 41]. If confirmed, this association points to CTCs as a factor informing on tumor‐immune interaction and suggests that CTC counts should be explored as a potential biomarker for effect of ICI therapy. In the ALICE trial, the benefit of atezolizumab was confined to patients with above‐median TIS [30] and appeared particularly strong for patients within the atezo‐chemo arm with a TIS above the 66th percentile (HR 0.42, 95% CI 0.17–1.03), which is a cutoff also reported for efficacy of PD‐1 blockers in other tumor forms [41, 42].

In our ICI‐treated cohorts, baseline CTCs were not prognostic for PFS, but sample size was limited, and we find the expected negative OS pattern in line with data from conventional chemo‐ or endocrine therapy [13, 14]. Prognostic data on CTCs with the CellSearch assay in mBC patients treated with ICI are limited. Inferior survival in patients with increased CTCs prior to immunotherapy has been shown in a cohort of 104 non‐small cell lung cancer patients [43]. The same trial also reported six times higher durable response rates for patients with negative samples 4–6 weeks into therapy compared to CTC‐positive patients. Similarly, our data suggest that CTC counts at week 4 were predictive of survival outcomes.

Our analyses were restricted by the low proportion of TNBC patients with baseline counts of ≥ 5 CTCs per 7.5 mL with only 3/21 (14%) vs. 17/34 (50%) among the HR^+^ patients. Interestingly, this was also apparent when grouped by intrinsic subtype with the lowest counts within the basal‐like population and the highest in patients with luminal A disease. Downregulation of epithelial markers secondary to epithelial–mesenchymal transition (EMT) is common in CTCs, with the highest proportion in TNBC and the lowest within the HR^+^ HER2^−^ subtype [44]. This may reduce sensitivity from EpCAM‐based CTC isolation [45, 46]; thus, a continuum of EMT phenotypes may explain some of the disparity in CTC counts between the more differentiated luminal A subtype and the basal‐like tumors. In the large pooled analysis by Bidard et al. [14], they reported a concordant proportion (51.1%) of patients with ≥ 5 CTCs per 7.5 mL within the HR^+^ subtype, but with a higher fraction (44.2%) having ≥ 5 CTCs per 7.5 mL in the TNBC population compared to our study. Thus, our skewed distribution within the TNBC cohort may be a random finding caused by a limited sample size.

We observed PD‐L1 expression on CTCs from 6 of 17 CTC‐positive patients (35%) at baseline. Data from a limited number of mBC cohorts analyzed by the CellSearch system have been published. In the first report by Mazel et al. [25], PD‐L1 expression was observed in 11 of 16 CTC‐positive patients (69%). In a larger cohort reported by the same group, 26 of 57 (46%) CTC‐positive patients had PD‐L1 expression [47]. Darga et al. [48] analyzed samples from a large cohort of 124 mBC patients detecting CTCs in 82 patients and PD‐L1 expression in 30 of these (37%). Thus, our results are in the range of what has been reported by others. Different PD‐L1 antibodies and a lack of standardized scoring systems are sources of variability. To ensure specificity, a 2+ intensity cutoff for positivity in a minimum of 10% of CTCs was set. A dim +1 signal in the PD‐L1 channel with an uncertain biological significance was frequent, and exclusion of all of these samples may have reduced sensitivity.

The CTC PD‐L1 positivity was not associated with the expression of PD‐L1 on tumor‐infiltrating immune cells, PD‐L1 gene expression or TIS in matched tumor biopsies in our analysis. Furthermore, only 2/6 patients with CTCs and PD‐L1^+^ biopsies had PD‐L1^+^ CTCs. This is in line with Jacot et al. [47] who found no correlation between CTC PD‐L1 expression and tumor PD‐L1 status by IHC in 56 mBC patients. Similar findings have been reported in NSCLC [49].

Zhou et al. [50] reported a predictive value of high PD‐L1 expression on CTCs and PFS in mBC patients treated with PD‐1 inhibitors. However, this was a peptide‐based CTC assay, and the predictive value of the CellSearch‐based CXC assay in mBC patients treated with ICI is still not known. Although the number of patients with PD‐L1^+^ CTCs in our dataset was too small for subset assessment, PD‐L1 expression on CTCs was associated with poor OS in the total cohort. This finding is in line with data from other publications [51].

Oligoprogressive disease is common in patients with acquired resistance to ICI and is associated with superior post‐progression survival compared to widespread systemic progression [52, 53, 54]. There is evidence suggesting that localized therapy and continuation of ICI may be effective in oligoprogressive disease [55, 56]. Interestingly, the two HR^+^ patients with acquired resistance to ipi/nivo‐only progressed without detectable CTCs and developed new lesions in immunologically privileged sites [57, 58]. Similarly, HR^+^ patients progressing without detectable CTCs in the ipi/nivo‐chemo cohort had a superior OS pattern. This suggests that CTC status may add clinically important information also at progression. In further studies, it would be of interest to investigate whether a lack of CTCs at progression on ICI‐containing regimes may be predictive of benefit from continuation of ICI.

## 5 Conclusions

This study demonstrates that CTC analyses during ICI therapy may give clinically relevant outcome information to some extent different from treatment with chemotherapy alone, although the interpretation should be cautious due to the limited number of samples analyzed. Furthermore, the tumor biology and immune composition may be reflected in the CTC status, which in our study was associated with molecular subtype and markers of tumor immune activation. PD‐L1 expression on CTCs showed no clear correlation with PD‐L1 status in tumor, but the overall CTC count correlated with immune genes reported to predict ICI efficacy. Beyond the prognostic information from CTC status, the results suggest that CTC detection may be further explored also as a potential biomarker for benefit from ICI therapy.

## Conflict of interest

JAK has in the last 5 years received research support from NanoString, Bristol Myers Squibb, F. Hoffmann‐La Roche, and NEC OncoImmunity and has previously received advisory board/lecture honoraria from pharmaceutical companies, including Bristol Myers Squibb. BG has received honoraria for advisory boards from Roche, Eli Lilly, Gilead, Daiichi Sankyo, and Pierre Fabre. HGR has received research support from NanoString and Illumina.

## Author contributions

Project conceptualization and funding: BN, JAK, and HGR. Coordinating investigator of the clinical trials responsible for design, funding, and regulatory approvals: JAK. Data analysis: NKA and AHR. Data interpretation: NKA, AHR, BN, JAK, and EB. CTC data collection and coordination: EB, CBS, HGR, AHR, NKA, BG, MS, ON, and RRM. Project pathologists: EB, HGR, ØG, and JL. Project statistician: RSF. Manuscript writing: NKA, BNA, and JAK with contribution from all authors. All authors have approved the final manuscript.

## Peer review

The peer review history for this article is available at https://www.webofscience.com/api/gateway/wos/peer‐review/10.1002/1878‐0261.13675.

## Supporting information


**Fig. S1.** CTC PD‐L1 analysis with the D8T4X/CXC assay.

**Fig. S2.** Comparison of CTC enumeration by analysis kit.

**Fig. S3.** Differential expression of gene expression signatures.

**Fig. S4.** Progression‐free survival by baseline CTC count.

**Fig. S5.** Survival outcomes by week 4 CTC count (≥ 5 CTCs per 7.5 mL).

**Fig. S6.** Combined analysis of survival outcomes in all patients by baseline and week 4 CTC counts.

**Fig. S7.** HR^+^ cohorts post progression survival by end of treatment CTCs.

**Table S1.** Baseline demographics and patient characteristics.
**Table S2.** Baseline CTCs counts vs. patient demographics and disease characteristics.

## Data Availability

The data that support the findings of this study are available upon reasonable request. Requests should be made to the corresponding author (bna@ous-hf.no) and will be reviewed by the study team. Data from this study are subject to patient confidentiality, and the transfer of data or materials will require approval from the Regional Committee for Medical and Health Research Ethics South‐East Norway.
