# Supplementary material for: Circulating tumor cells in metastatic breast cancer patients treated with immune checkpoint inhibitors – a biomarker analysis of the ALICE and ICON trials
Source: Mol Oncol. 2024 Jul 8;19(7):2092–108. doi: 10.1002/1878-0261.13675 (PMC12234385; doi:10.1002/1878-0261.13675)
Supplement: Supplementary file 1 — Fig. S1. CTC PD‐L1 analysis with the D8T4X/CXC assay. [file MOL2-19-2092-s008.pdf]

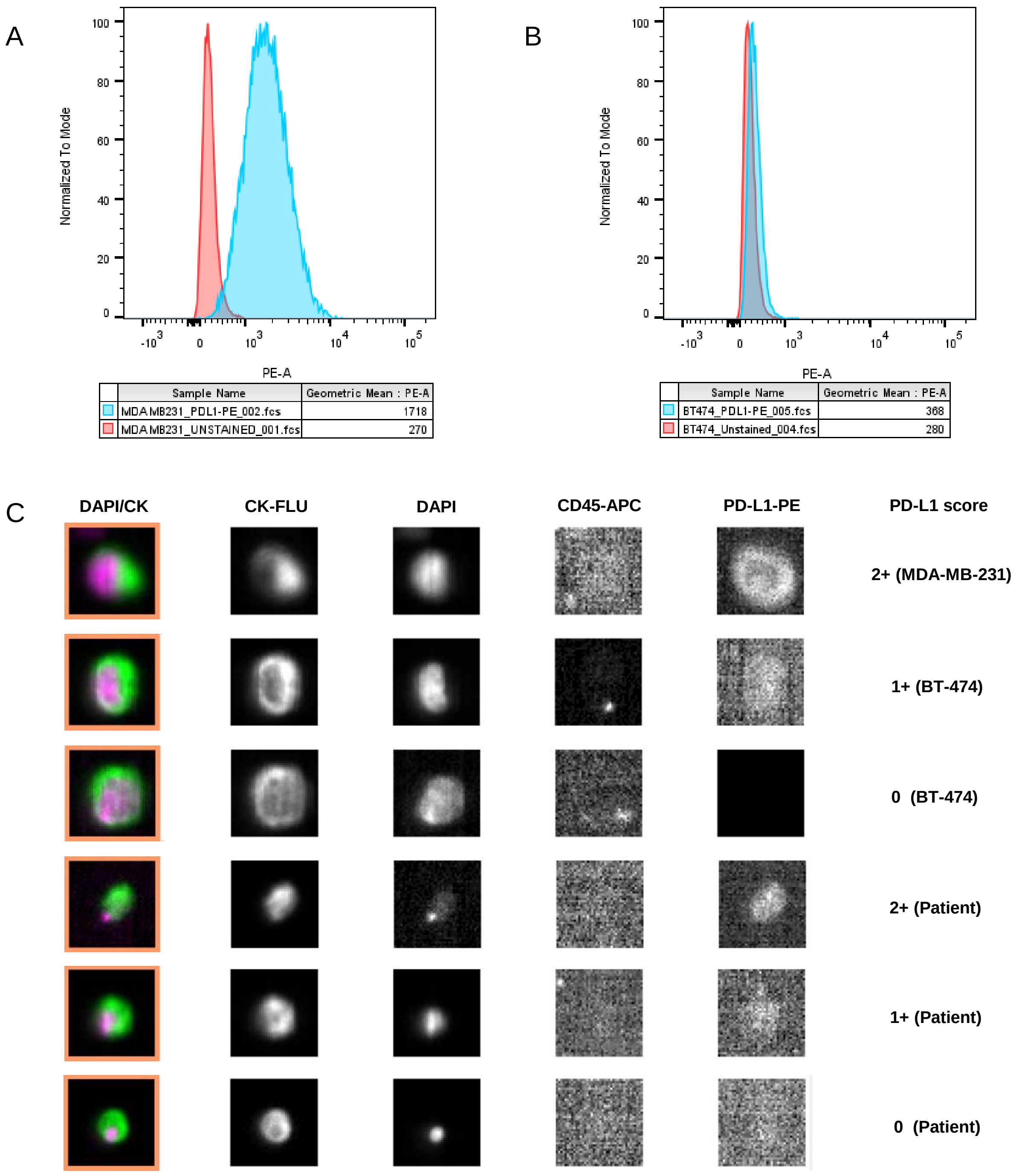

Figure S1 | CTC PD-L1 analysis with the D8T4X/CXC assay

The MDA-MB-231 and BT-474 breast cancer cell lines were used as positive and negative controls for PD-L1 expression with the D8T4X/CXC assay. Panel **A** and **B** show flow cytometry data of PD-L1 expression with the PE-conjugated D8T4X antibody, confirming PD-L1 expression in the positive cell line (MDA-MB-231) (**A**) and the absence of PD-L1 expression in the negative cell line (BT474) (**B**). Panel **C** presents the 0-2 scale of PD-L1 intensity scoring of CTCs analyzed with the D8T4X/CXC assay. The selected CTCs show the spectrum of staining both from the MDA-MB-231 and BT-474 cell lines and from patients. The scoring system was defined as: no signal in the PE-channel (0). Dim signal but not stronger than the brightest signal also observed in the PD-L1 negative BT474 cell line (1+). Brighter signal than any CTC in the negative cell line (2+).

Abbreviations: CTCs, circulating tumor cells; PD-L1, programmed death-ligand 1; PE, phycoerythrin
