# Supplementary material for: Circulating tumor cells in metastatic breast cancer patients treated with immune checkpoint inhibitors – a biomarker analysis of the ALICE and ICON trials
Source: Mol Oncol. 2024 Jul 8;19(7):2092–108. doi: 10.1002/1878-0261.13675 (PMC12234385; doi:10.1002/1878-0261.13675)
Supplement: Supplementary file 2 — Fig. S2. Comparison of CTC enumeration by analysis kit. [file MOL2-19-2092-s005.pdf]

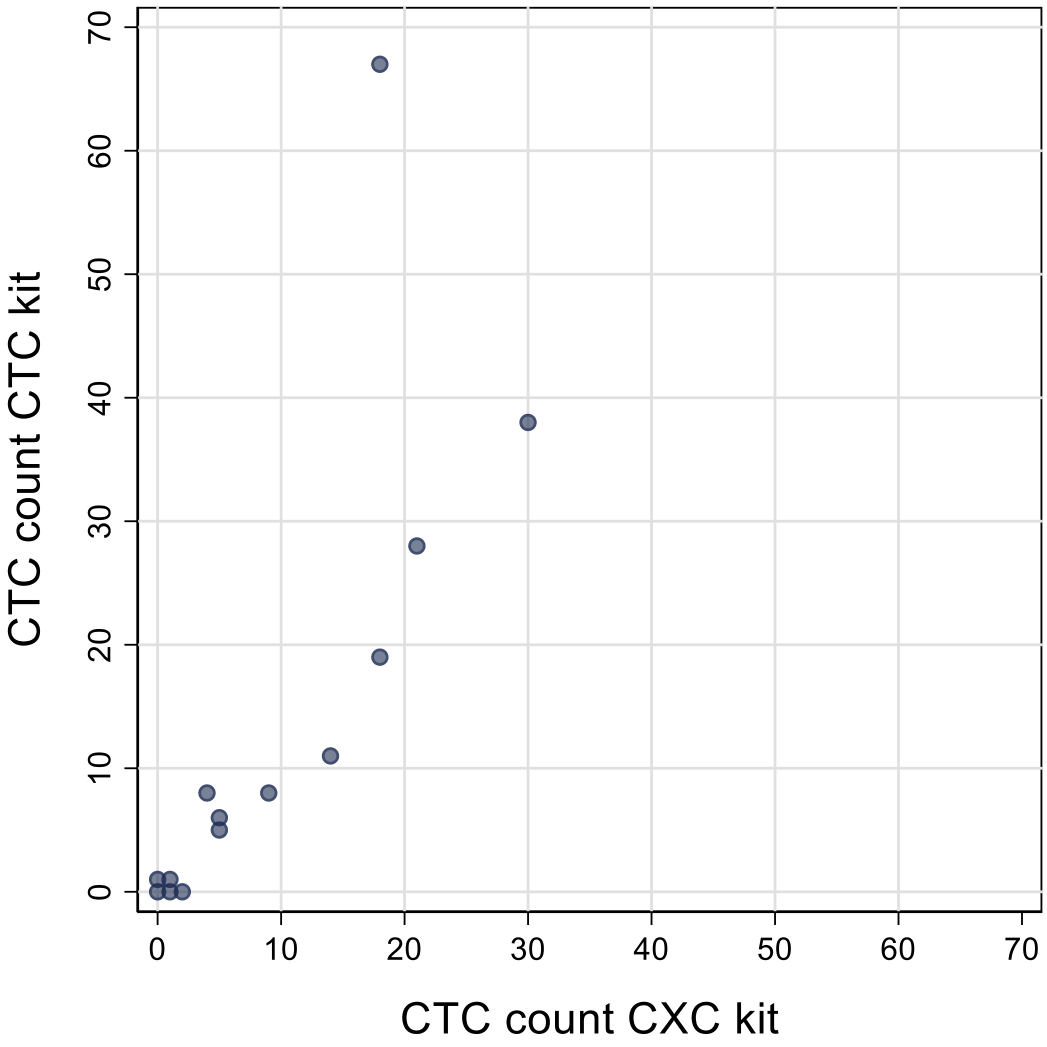

Figure S2 | Comparison of CTC enumeration by analysis kit
The figure presents a comparison of CTC counts by the CXC kit versus the CTC kit in 14 patients sampled with two separate samples of whole blood at the same time point. One sample was processed with the research only CXC kit (x-axis) and the other processed with the standard CTC kit (y-axis).
