# Supplementary material for: Circulating tumor cells in metastatic breast cancer patients treated with immune checkpoint inhibitors – a biomarker analysis of the ALICE and ICON trials
Source: Mol Oncol. 2024 Jul 8;19(7):2092–108. doi: 10.1002/1878-0261.13675 (PMC12234385; doi:10.1002/1878-0261.13675)
Supplement: Supplementary file 3 — Fig. S3. Differential expression of gene expression signatures. [file MOL2-19-2092-s004.pdf]

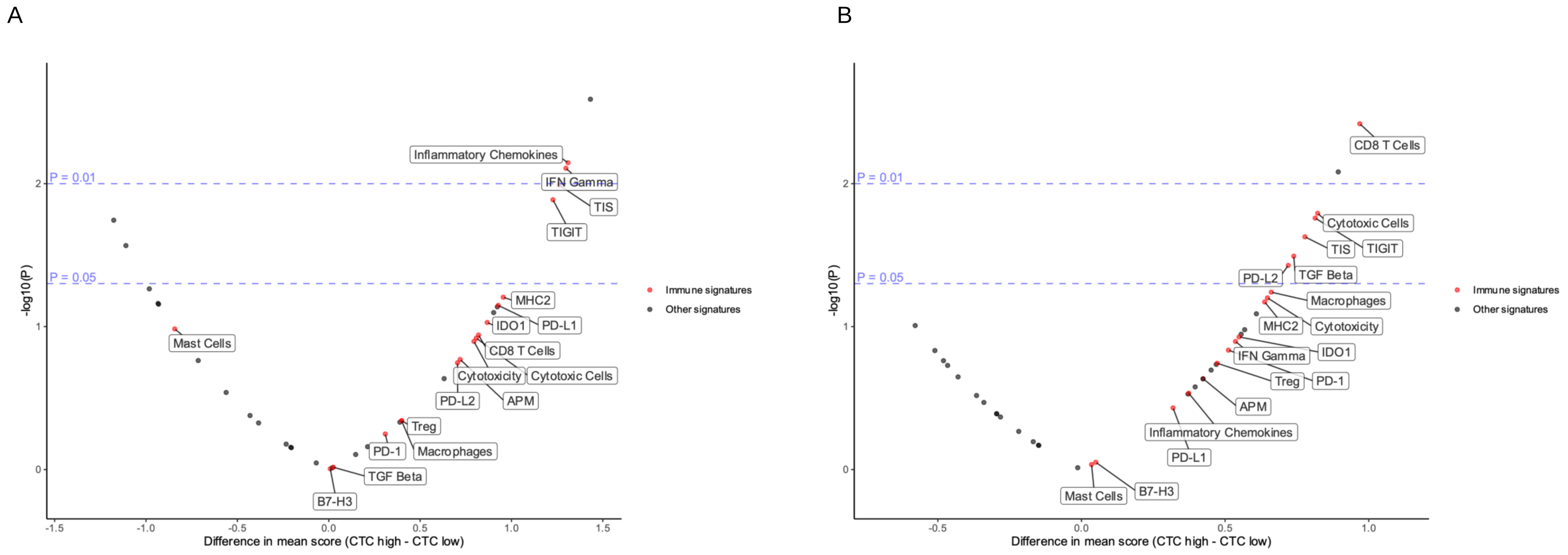

Figure S3 | Differential expression of gene expression signatures

The differential expression of gene signatures from the NanoString BC360 kit by CTC levels at baseline with a ≥2 CTCs/7.5 mL cutoff was analyzed. The BC360 kit includes 42 different gene expression signatures with 18 of the signatures related to immune function. The figure presents volcano plots of expression of all signatures with the 18 immune-related signatures highlighted in red. Panel **A** presents the differential expression of signatures within the TNBC population by CTC low (*n* = 15) versus CTC high (*n* = 5) patients. Panel **B** presents the HR^+ population by CTC low (*n* = 15) versus CTC high (*n* = 18) patients. In patients with more than one sample analyzed with the NanoString BC360 assay, the most recent sample available was used. *P* values are calculated with the *t* test.

Abbreviations: CTC, circulating tumor cells; TNBC, triple-negative breast cancer; HR^+, hormone receptor-positive; TIS, Tumor Inflammation Signature; TGF-beta, transforming growth factor-β; PD-L1, programmed death-ligand 1; PD-L2, programmed cell death ligand-2; APM, antigen processing machinery; Treg, regulatory T cells
