# Supplementary material for: Circulating tumor cells in metastatic breast cancer patients treated with immune checkpoint inhibitors – a biomarker analysis of the ALICE and ICON trials
Source: Mol Oncol. 2024 Jul 8;19(7):2092–108. doi: 10.1002/1878-0261.13675 (PMC12234385; doi:10.1002/1878-0261.13675)
Supplement: Supplementary file 4 — Fig. S4. Progression‐free survival by baseline CTC count. [file MOL2-19-2092-s006.pdf]

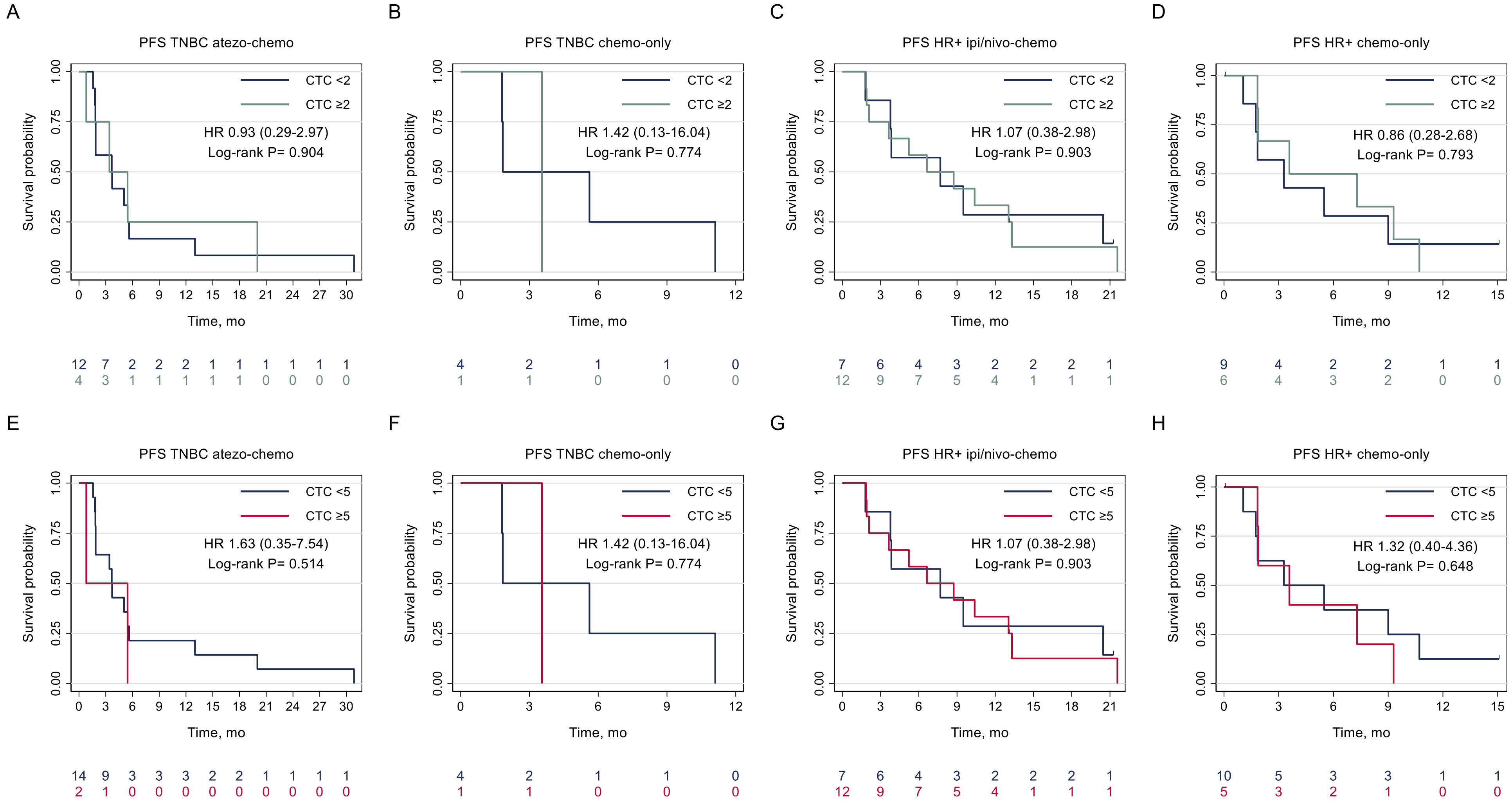

Figure S4 | Progression-free survival by baseline CTC count

The figure presents Kaplan-Meier plots of PFS by baseline CTC count with the ≥2 CTCs/7.5 mL cutoff in each of the four treatment cohorts in **A**-**D** and by the ≥5 CTCs/7.5 mL cutoff in **E**-**H**.

Abbreviations: CTC, circulating tumor cells; TNBC, triple-negative breast cancer; HR+, hormone receptor-positive; PFS, progression-free survival; HR, hazard ratio; atezo, atezolizumab; ipi, ipilimumab; nivo, nivolumab
