# Supplementary material for: Circulating tumor cells in metastatic breast cancer patients treated with immune checkpoint inhibitors – a biomarker analysis of the ALICE and ICON trials
Source: Mol Oncol. 2024 Jul 8;19(7):2092–108. doi: 10.1002/1878-0261.13675 (PMC12234385; doi:10.1002/1878-0261.13675)
Supplement: Supplementary file 5 — Fig. S5. Survival outcomes by week 4 CTC count (≥ 5 CTCs per 7.5 mL). [file MOL2-19-2092-s002.pdf]

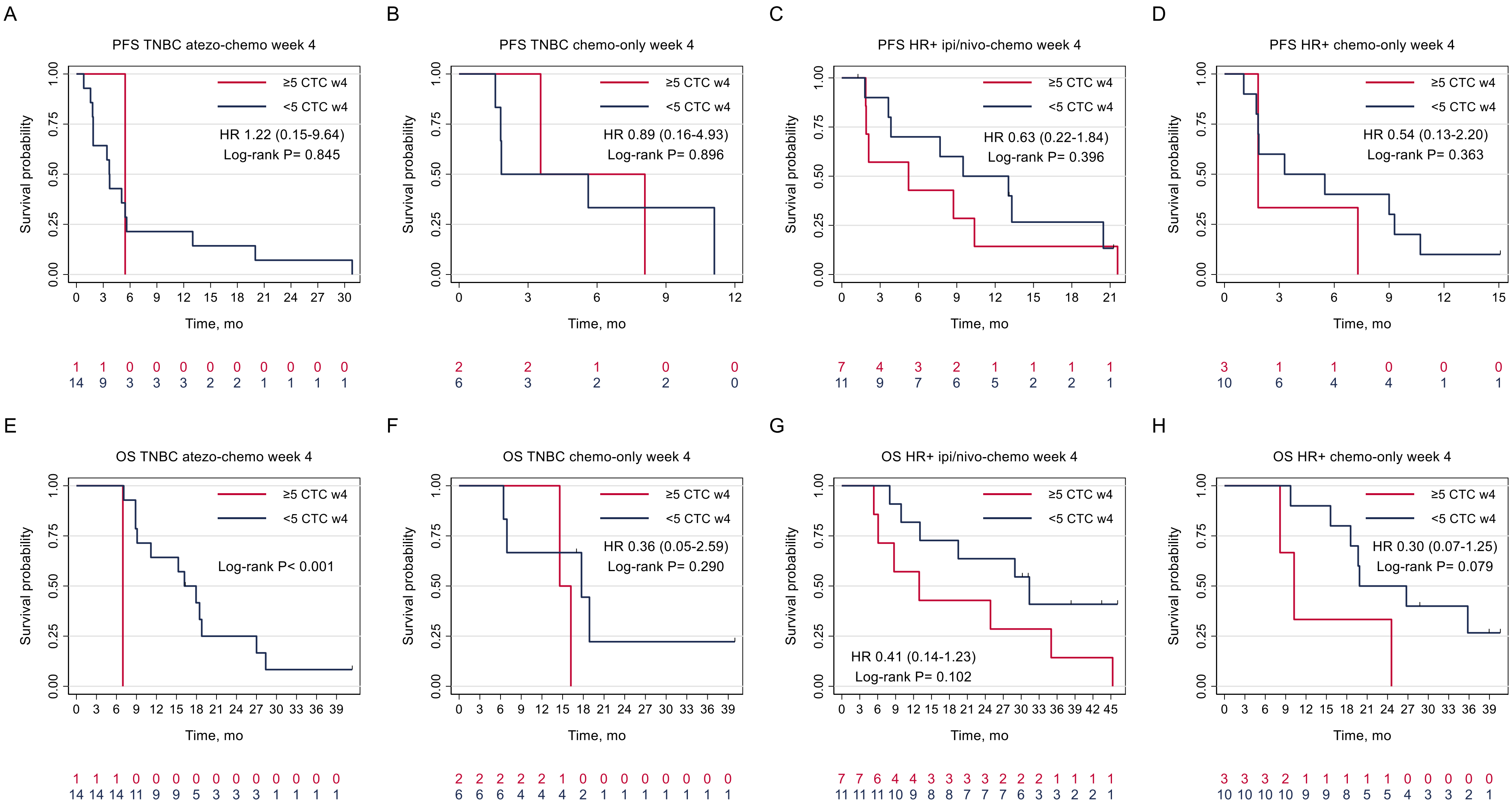

Figure S5 | Survival outcomes by week 4 CTC count (≥5 CTCs/7.5 mL)

The figure presents Kaplan-Meier plots of PFS by CTCs measured at week 4 by the ≥5 CTCs/7.5 mL cutoff in each of the four treatment cohorts in **A-D**. OS in each cohort is presented in **E-H**.

Abbreviations: CTC, circulating tumor cells; TNBC, triple-negative breast cancer; HR+, hormone receptor-positive; PFS, progression-free survival; OS, overall survival; HR, hazard ratio; w4, week 4; atezo, atezolizumab; ipi, ipilimumab; nivo, nivolumab
