# Supplementary material for: Circulating tumor cells in metastatic breast cancer patients treated with immune checkpoint inhibitors – a biomarker analysis of the ALICE and ICON trials
Source: Mol Oncol. 2024 Jul 8;19(7):2092–108. doi: 10.1002/1878-0261.13675 (PMC12234385; doi:10.1002/1878-0261.13675)
Supplement: Supplementary file 6 — Fig. S6. Combined analysis of survival outcomes in all patients by baseline and week 4 CTC counts. [file MOL2-19-2092-s003.pdf]

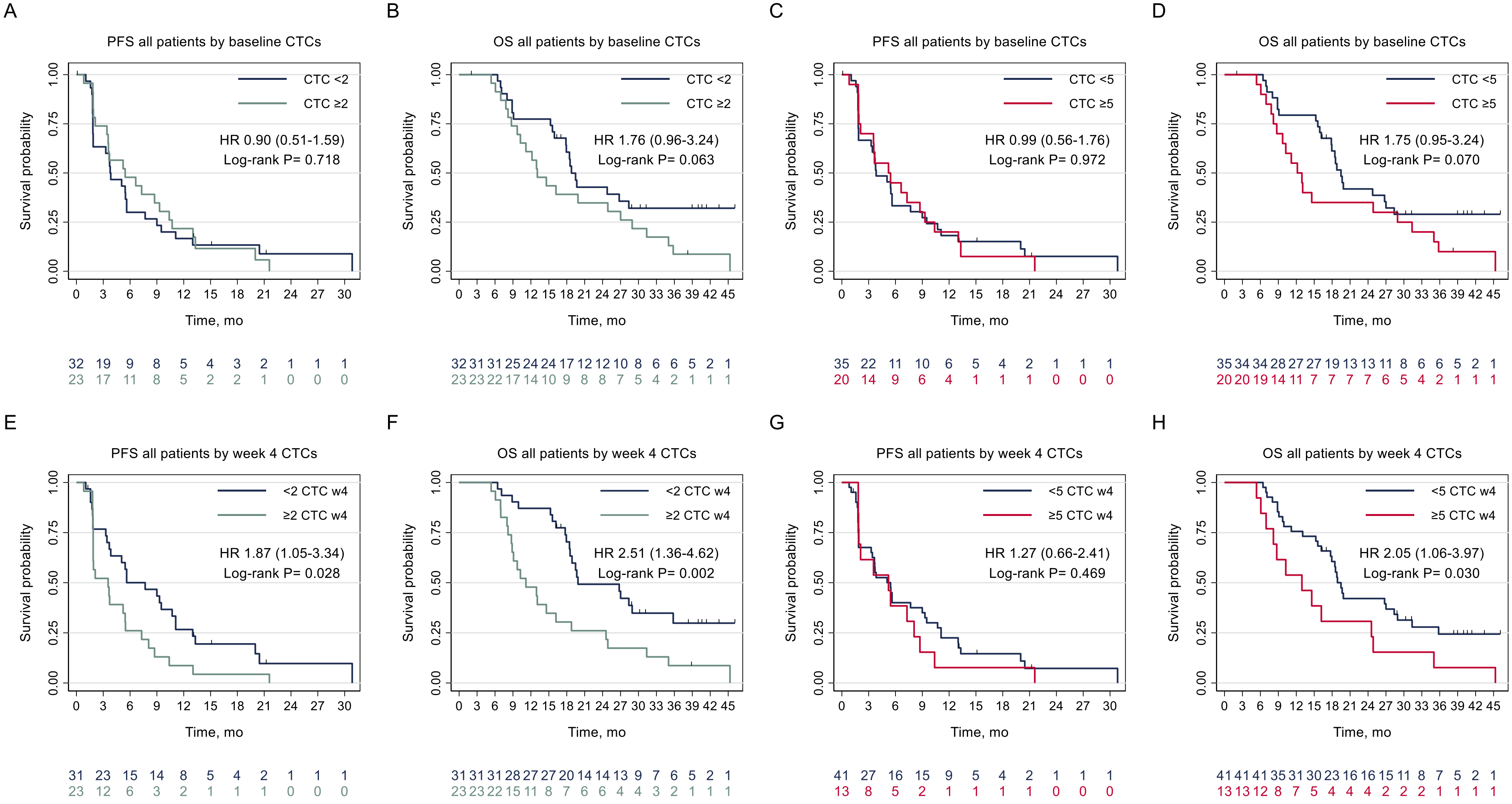

Figure S6 | Combined analysis of survival outcomes in all patients by baseline and week 4 CTC counts

The figure presents Kaplan-Meier plots of survival outcomes for all patients by baseline (**A-D**) and week 4 (**E-H**) CTC counts. Panel **A** presents PFS and **B** OS by baseline CTC counts with the ≥2 CTCs/7.5 mL cutoff. Panel **C** presents PFS and **D** OS by baseline CTC counts with the ≥5 CTCs/7.5 mL cutoff. PFS and OS by the ≥2 CTCs/7.5 mL cutoff by week 4 are presented in **E** and **F**. PFS and OS by the ≥5 CTCs/7.5 mL cutoff by week 4 are presented in **G** and **H**.

Abbreviations: CTC, circulating tumor cells; PFS, progression-free survival; OS, overall survival; HR, hazard ratio; w4, week 4
