# Supplementary material for: Circulating tumor cells in metastatic breast cancer patients treated with immune checkpoint inhibitors – a biomarker analysis of the ALICE and ICON trials
Source: Mol Oncol. 2024 Jul 8;19(7):2092–108. doi: 10.1002/1878-0261.13675 (PMC12234385; doi:10.1002/1878-0261.13675)
Supplement: Supplementary file 7 — Fig. S7. HR+ cohorts post progression survival by end of treatment CTCs. [file MOL2-19-2092-s007.pdf]

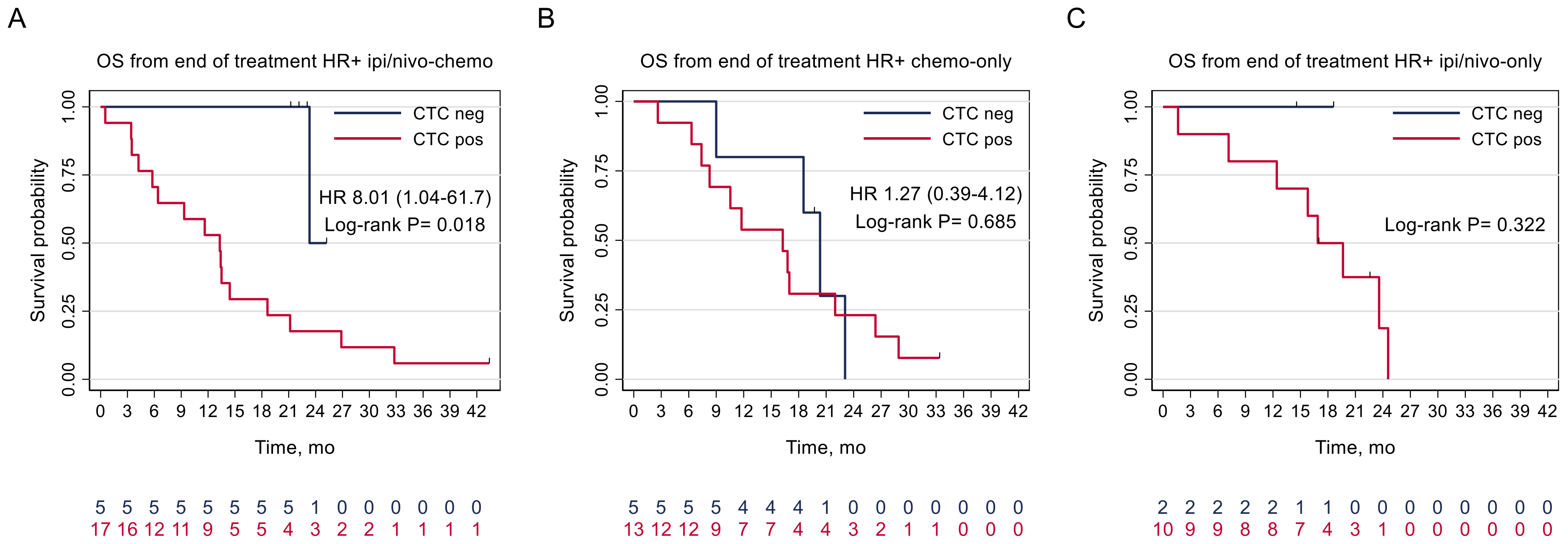

Figure S7 | HR⁺ cohorts post-progression survival by end of treatment CTCs

Kaplan-Meier plots of overall survival by CTC presence (≥1 CTCs/7.5 mL) in EOT samples calculated from the EOT time point in the HR$^+$ cohorts. The HR$^+$ ipi/nivo-chemo cohort is presented in **A**, HR$^+$ chemo-only in **B** and HR$^+$ ipi/nivo-only in **C**. Two patients in both the ipi/nivo-chemo and chemo-only cohorts without progression at EOT were excluded from the analysis.

Abbreviations: CTC, circulating tumor cells; HR+, hormone receptor-positive; OS, overall survival; HR, hazard ratio; EOT, end of treatment; ipi, ipilimumab; nivo, nivolumab
