# Supplementary material for: Circulating tumor cells in metastatic breast cancer patients treated with immune checkpoint inhibitors – a biomarker analysis of the ALICE and ICON trials
Source: Mol Oncol. 2024 Jul 8;19(7):2092–108. doi: 10.1002/1878-0261.13675 (PMC12234385; doi:10.1002/1878-0261.13675)
Supplement: Supplementary file 8 — Table S1. Baseline demographics and patient characteristics. Table S2. Baseline CTCs counts vs. patient demographics and disease characteristics. [file MOL2-19-2092-s001.pdf]

	TNBC atezo-chemo (*n* = 20)	TNBC chemo-only (*n* = 12)	HR+ ipi/nivo-chemo (*n* = 28)	HR+ chemo-only (*n* = 22)	HR+ ipi/nivo-only (*n* = 12)
**Age, years (IQR)**	59 (54-63)	55 (38-60)	52 (48-58)	55 (49-62)	57 (50-63)
**ECOG performance status, *n* (%)**					
0	14 (70)	10 (83)	13 (46)	14 (64)	10 (83)
1	6 (30)	2 (17)	15 (54)	8 (36)	2 (17)
**Time with stage IV disease, mo (IQR)**	2 (1-5)	3 (1-21)	28 (19-43)	24 (19-50)	45 (30-73)
**De novo metastatic disease, *n* (%)**	4 (20)	3 (25)	7 (25)	6 (27)	4 (33)
**Sites of metastases *n* (%)**					
Bone metastases	8 (40)	5 (42)	26 (93)	18 (82)	11 (92)
Liver metastases	7 (35)	4 (33)	19 (68)	19 (86)	11 (92)
Lymph nodes	9 (45)	7 (58)	10 (36)	11 (50)	7 (58)
Lung metastases	8 (40)	7 (58)	11 (39)	3 (14)	2 (17)
> 3 sites of metastases	2 (10)	1 (8)	9 (32)	4 (18)	3 (25)
**Previous lines of metastatic chemo, *n* (%)**					
0*	16 (80)	6 (50)	22 (79)	12 (55)	0
1	4 (20)	6 (50)	6 (21)	10 (45)	6 (50)
2	0	0	0	0	6 (50)
**Intrinsic breast cancer subtype, *n* (%)**					
Luminal A	0	1 (8)	7 (25)	3 (14)	1 (8)
Luminal B	1 (5)	1 (8)	18 (64)	16 (73)	9 (75)
HER2 enriched	4 (20)	1 (8)	3 (11)	2 (9)	1 (8)
Basal	15 (75)	8 (67)	0	0	0
Missing	0	1 (8)	0	1 (5)	1(8)
**PD-L1 status (IHC), *n* (%)**					
Positive	8 (40)	7 (58)	5 (18)	2 (9)	2 (17)
Negative	12 (60)	5 (42)	23 (82)	20 (91)	10 (83)
**TIL score, *n* (%)**					
High (2/3+)	4 (20)	4 (33)	1 (4)	3 (14)	3 (25)
Low (0/1+)	16 (80)	8 (67)	26 (93)	19 (86)	9 (75)
Missing	0	0	1 (4)	0	0
**Baseline LDH (xULN), median (IQR)**	1.0 (0.8-1.2)	0.9 (0.8-1.1)	1.3 (1.0-1.5)	1.0 (0.9-1.1)	1.0 (0.9-1.2)
**Baseline CRP (xULN), median (IQR)**	1.4 (0.6-3.2)	0.6 (0.3-0.9)	1.7 (0.8-3.8)	0.6 (0.2-2.1)	0.4 (0.3-1.3)

Table S1 | Baseline demographics and patient characteristics

Continuous measures are presented as median (IQR) and categorical data as *n* (%). In patients with more than one sample analyzed, the intrinsic subtype and PD-L1 status by IHC were assessed in the most recent archival sample. The TIL score was assessed in a study biopsy if sufficient material, otherwise in the most recent archival biopsy available. Lactate dehydrogenase and C-reactive protein levels are presented relative to the upper limit of normal. *Patients who had started or received first-line treatment with an anthracycline-based regimen without disease progression were classified as continuation of first-line treatment. Abbreviations: HR^+, hormone receptor positive; HER2, human epidermal growth factor receptor 2; TNBC, triple-negative breast cancer; ECOG, Eastern Cooperative Oncology Group; PD-L1, programmed death-ligand 1; IHC, immunohistochemistry; TIL, tumor-infiltrating lymphocytes; CRP, C-reactive protein; LDH, lactate dehydrogenase; ULN, upper limit of normal

	TNBC cohort baseline (*N*= 21)						HR+ cohort baseline (*N*= 34)					
	**CTC <2 (*n* = 16)**	**≥2 CTC (*n* = 5)**	***P* value**	**CTC <5 (*n* = 18)**	**≥5 CTC (*n* = 3)**	***P* value**	**CTC <2 (*n* = 16)**	**≥2 CTC (*n* = 18)**	***P* value**	**CTC <5 (*n* = 17)**	**≥5 CTC (*n* = 17)**	***P* value**
**Age, years (IQR)**	59 (42-64)	58 (56-59)	0.80	59 (43-65)	58 (33-59)	0.37	55 (50-63)	53 (48-59)	0.41	55 (50-62)	53 (50-59)	0.77
**ECOG performance status, *n* (%)**												
0	13 (81)	4 (80)	0.95	15 (83)	2 (67)	0.50	13 (81)	6 (33)	**0.007**	14 (82)	5 (29)	**0.005**
1	3 (19)	1 (20)		3 (17)	1 (33)		3 (19)	12 (67)		3 (18)	12 (71)	
**Time with stage IV disease, mo (IQR)**	1.4 (0.9-4.9)	0.6 (0.6-1.9)	0.32	1 (1-4)	2 (1-13)	0.65	34 (25-66)	22 (17-30)	**0.014**	34 (24-66)	23 (17-30)	**0.040**
**De novo metastatic disease, *n* (%)**	4 (25)	2 (40)	0.60	5 (28)	1 (33)	1.00	4 (25)	4 (22)	1.00	4 (24)	4 (24)	1.00
**Sites of metastases *n* (%)**												
Bone metastases	5 (31)	4 (80)	0.12	6 (33)	3 (100)	0.063	12 (75)	18 (100)	**0.039**	13 (76)	17 (100)	0.10
Liver metastases	4 (25)	2 (40)	0.60	5 (28)	1 (33)	1.00	10 (62)	14 (78)	0.46	11 (65)	13 (76)	0.71
Lymph nodes	9 (56)	4 (80)	0.61	11 (61)	2 (67)	1.00	8 (50)	6 (33)	0.49	8 (47)	6 (35)	0.73
Lung metastases	8 (50)	1 (20)	0.34	9 (50)	0	0.23	9 (56)	3 (17)	**0.030**	9 (53)	3 (18)	0.071
> 3 sites of metastases	1 (6)	1 (20)	0.43	2 (11)	0	1.00	5 (31)	5 (28)	1.0	5 (29)	5 (29)	1.00
**Previous lines of metastatic chemo, *n* (%)**												
0*	11 (69)	5 (100)	0.28	13 (72)	3 (100)	0.55	11 (69)	10 (56)	0.50	12 (71)	9 (53)	0.48
1	5 (31)	0		5 (28)	0		5 (31)	8 (44)		5 (29)	8 (47)	
**Intrinsic breast cancer subtype, *n* (%)**												
Luminal A	0	0	0.65	0	0	1.00	2 (12)	6 (33)	0.40	2 (12)	6 (35)	0.34
Luminal B	1 (6)	0		1 (6)	0		11 (69)	10 (56)		12 (71)	9 (53)	
HER2 enriched	4 (25)	0		4 (22)	0		2 (12)	2 (11)		2 (12)	2 (12)	
Basal	10 (62)	5 (100)		12 (67)	3 (100)		0	0		0	0	
Missing	1 (6)	0		1 (6)	0		1 (6)	0		1 (6)	0	
**Baseline LDH (xULN), median (IQR)**	0.9 (0.8-1.2)	1.2 (1.0-1.4)	0.17	1.0 (0.8-1.3)	1.0 (0.9-1.4)	0.65	0.9 (0.9-1.3)	1.2 (1.0-1.5)	0.055	0.9 (0.9-1.2)	1.3 (1.1-1.5)	**0.016**
**Baseline CRP (xULN), median (IQR)**	0.6 (0.1-1.8)	3.8 (0.9-6.8)	0.062	0.8 (0.1-2.8)	0.9 (0.6-3.8)	0.65	0.7 (0.3-2.3)	2.0 (0.8-6.3)	**0.034**	0.7 (0.1-2.3)	2.3 (0.9-6.3)	**0.010**

Table S2 | Baseline CTCs counts versus patient demographics and disease characteristics

The table presents patients demographics and disease characteristics by baseline CTC counts in the TNBC and HR^+ population by both the ≥2 CTCs/7.5 mL and ≥5 CTCs/7.5 mL cutoffs. Categorical data are presented as *n* (%) with *P* values calculated by the Fisher's exact test. Continuous data are presented as the median (IQR) with *P* values calculated by the Wilcoxon rank-sum test. In patients with more than one sample analyzed, the intrinsic subtype was assessed in the most recent archival sample. Lactate dehydrogenase and C-reactive protein levels are presented relative to the upper limit of normal. * Patients who had started or received first-line treatment with an anthracycline-based regimen without disease progression were classified as continuation of first-line treatment. Abbreviations: TNBC, triple-negative breast cancer; HR^+, hormone receptor positive; ECOG, Eastern Cooperative Oncology Group; CRP, C-reactive protein; LDH, lactate dehydrogenase; ULN, upper limit of normal
